# Metabolomic-genomic prediction realizes small increases in accuracy of estimated breeding values for daily gain in pigs

**DOI:** 10.1186/s12711-025-00972-4

**Published:** 2025-05-21

**Authors:** Xiangyu Guo, Pernille Sarup, Anders Bay Nord, Mark Henryon, Tage Ostersen, Ole F. Christensen

**Affiliations:** 1 Danish Pig Research Centre, Danish Agriculture & Food Council, 1609 Copenhagen V, Denmark; 2 Center for Quantitative Genetics and Genomics, Aarhus University, 8000 Aarhus C, Denmark; 3 Nordic Seed A/S, 8300 Odder, Denmark; 4 Swedish NMR Centre, University of Gothenburg, Box 465, 40530 Göteborg, Sweden

## Abstract

**Background:**

Metabolomic profiling of blood samples can be done on selection candidates and could be a valuable information source for genetic evaluation of pigs. We hypothesized that integrating metabolomic data from pigs without individual phenotypes into the metabolomic-genomic best linear unbiased prediction (MGBLUP) model would generate estimated breeding values (EBVs) with a higher accuracy compared to what would be obtained without metabolomic data. We tested this hypothesis by predicting breeding values for average daily gain (ADG) using phenotypic, genomic, and metabolomic data. MGBLUP models were fitted to average daily gain of 8174 Duroc pigs that were genotyped and profiled for metabolomic features. Approximately half the pigs were males from a test station and the other half were females from breeding herds. Variance components were estimated, and we employed two validation schemes: test station to breeding herd validation and fivefold cross-validation. Accuracies of EBVs in the validation population were computed by combining results on predictive abilities with results on increases in accuracies from the linear regression method.

**Results:**

Parameter estimates from MGBLUP showed a direct heritability of ADG of 0.15, a proportion of variance explained by metabolomic features of 0.18, and a heritability of metabolomic intensities of 0.14, together resulting in a total heritability of 0.17. Thus, the majority of the heritability was not mediated by the metabolome. For the test station to breeding herd validation, the accuracies of EBVs were 0.60 for genomic best linear unbiased prediction (GBLUP) with genotypes in validation population, 0.61 for MGBLUP with genotypes in validation population, 0.62 for MGBLUP with genotypes and metabolomic features in validation population, 0.72 for GBLUP with genotypes and phenotypes in validation population, and 0.74 for MGBLUP with genotypes, phenotypes and metabolomic features in validation population, whereas the corresponding numbers were 0.87, 0.87, 0.87, 0.91 and 0.92 for the fivefold cross-validation. Therefore, small increases in accuracies were observed when including metabolomic features.

**Conclusions:**

The inclusion of metabolomics data provided small improvements in the accuracy of genetic evaluations for average daily gain in pigs. Further work will be needed to investigate, e.g., alternative time points for blood sampling, metabolomics on samples of other tissues, and other traits.

**Supplementary Information:**

The online version contains supplementary material available at 10.1186/s12711-025-00972-4.

## Background

Genetic improvement in pig breeding relies on accurate prediction of breeding values for traits critical to pig production. Accurate prediction can be challenging when some selection candidates are not recorded for these traits (i.e., they do not have own phenotype for a trait). Selection candidates in pig breeding programs often lack own phenotypes for litter size, sow longevity, meat quality, and feed efficiency, because these traits either cannot be recorded before selection or are expensive to measure. Without own phenotypes, prediction of breeding values for each selection candidate relies on pedigree, genomic markers, and phenotypes of related animals or correlated traits. Recent animal breeding literature [1] has suggested the incorporation of diverse high-dimensional biological data as a possible avenue for increasing accuracies of estimated breeding values (EBVs) for young individuals and for such traits.

Metabolomics, i.e. the analysis of metabolites in biological entities, are a high-dimensional data resource that offers a dynamic perspective into an organism’s physiological state [2]. The blood metabolome provides a detailed profile of metabolites that reflects an animal's overall physiological state [3]. It serves as an invaluable resource for metabolomic studies in pigs, as it captures the dynamic exchange of metabolites between organs, offering a holistic snapshot of the organism’s metabolic state [4]. A previous study identified key metabolomic pathways and gene-metabolite interactions linked to pig growth, revealing the biological mechanisms underlying growth performance through integrated metabolomic and transcriptomic analysis [5]. Another study using proton nuclear magnetic resonance spectroscopy showed that blood metabolomic profiling is a powerful tool for predicting growth-related phenotypes, offering potential to increase accuracies of EBVs in pig breeding [6]. In pig breeding, metabolomic profiling of blood samples can be done on selection candidates and would therefore be a valuable information resource for genetic evaluation of traits for which no phenotypes are available on these individuals.

Addressing the inclusion of high dimensional omics data into genetic evaluation, Christensen et al. [7] introduced a joint model for phenotypes and omics data and methods for best linear unbiased prediction (BLUP) of breeding values. The model is based on the idea that all omics features can potentially be useful for genetic evaluation, through the construction of an omics similarity matrix between individuals, similar to the use of a marker-based genetic relationship matrix in the commonly used genomic best linear unbiased prediction (GBLUP) model [8–10]. Further, Guo et al. [11] adapted the model and methods of Christensen et al. [4] to combine phenotypic, genomic, and metabolomic data for breeding value prediction in barley, and named these the metabolomic-genomic model and metabolomic-genomic best linear unbiased prediction (MGBLUP). The results showed that for 4 out of 5 barley malting quality traits, about 50% of the increase in accuracy of EBVs that was obtained by having own phenotype could be obtained by having own metabolomic data instead. As metabolomic data increases the accuracy of EBVs in barley, it may also increase prediction accuracy in other species, including pigs.

Therefore, we hypothesized that integrating metabolomic data from pigs without individual phenotypes into the MGBLUP model would generate EBVs with an increase in accuracy, compared to what would be obtained without metabolomic data. We tested this hypothesis by assessing accuracies of EBVs for average daily gain (ADG) using phenotypic, genomic, and metabolomic data in MGBLUP. The linear regression (LR) method [12], which provides a semi-parametric framework for evaluating the accuracy and bias of EBV, was used to measure increases in accuracies of EBVs from adding additional information, which were combined with estimates of predictive abilities (correlation between corrected phenotypes and EBVs) to obtain empirical accuracies of EBVs.

## Methods

### Dataset

The comparison included data on 8174 Duroc pigs (4027 males, 4147 females) from the DanBred breeding system born between 2020 and 2022 (Table 1). Animals were recorded for ADG as part of routine evaluation. Males were assessed for ADG at a performance test station. Females were assessed in breeding herds.

**Table 1:** Number of male and female individuals born by year

Sex	2020	2021	2022	Total
Male	1237	2585	205	4027
Female	0	3850	297	4147

Each pig in the study had ADG in the bodyweight interval from 30 to 100 kg, genomic information, and records of Nuclear Magnetic Resonance (NMR) metabolomic features. Additional file 1 Table S1 presents the descriptive statistics for ADG in the study cohort. All pigs were genotyped for single-nucleotide polymorphism (SNP) markers using the 50 K GGP-Porcine Illumina Bead SNP as part of the DanBred breeding program. After quality control of SNP genotypes, we had 33,960 SNPs. The quality control involved the following steps and criteria: (1) SNPs had a known and unique position based on Sus Scrofa build 11.1; (2) SNPs were located on one of the 18 autosomes or on the X-chromosome; (3) SNPs had a minimum allele frequency above 0.001; (4) SNP genotypes did not deviate significantly from Hardy–Weinberg equilibrium (p-value < 0.0000001) [10]; (5) SNPs had a proportion of detected Mendelian errors below 0.001. Furthermore, for each genotyped pig, the minimum call rate was at least 80% and genotypes with parentage errors were re-genotyped. Imputation was done using FImpute 3 [13].

Blood samples for NMR analysis were collected from the pigs at the end date of ADG test. These samples were then sent to Swedish NMR Centre at University of Gothenburg, Sweden. NMR analyses were carried out in four batches, spanning mid-2021 to early 2023.

### Metabolomic data

#### NMR data

Plasma samples were thawed for 45 min before being mixed with equal amounts of buffer (75 mM sodium phosphate pH 7.4, 0.1% sodium azide, 20% v/v D_2_O, 0.08% TSP-d4) and then transferred to 5 mm SampleJet rack tubes. Sample preparation was done with the aid of a SamplePro Tube L liquid handling robot (Bruker BioSpin), where sample and NMR rack positions were kept cool at 2 °C during operation. ^1^H NMR data was acquired under Bruker In Vitro Diagnostics for research (IVDr; Bruker BioSpin) standard operating procedures [14], i.e. performing daily quality assurance (temperature, water suppression, shimming, quantification response), and acquiring one-dimensional (1D) Nuclear Overhauser Effect Spectroscopy, 1D Carr-Purcell-Meiboom-Gill-edited and two-dimensional J-resolved experiments for each sample. A 600 MHz Bruker Avance III HD spectrometer was used, equipped with a cooled SampleJet sample changer, where NMR-tube racks were kept at 6 °C before data acquisition.

#### Metabolomic features and NMR data processing

NMR data preprocessing ensures accurate analysis by correcting baseline variations, chemical shift alignment, and signal intensities from instrumental or sample-related factors. This standardizes spectral data, enabling reliable comparison of metabolic profiles across animals and batches and robust statistical analyses. In this investigation, our analysis centered on a comprehensive dataset of 32,697 NMR intensities for 8952 samples from the NMR analysis described above. We initially excluded 138 samples, either due to pronounced negative water peaks, suggesting errors in the NMR procedure, or because some animals had been sampled multiple times, resulting in duplicates. This left 8814 individuals, each with a single sample, for further processing. The NMR intensities were processed to facilitate their utilization in our study. The data spanned a frequency range from 0 to 10 ppm and was transformed using a bespoke MATLAB script designed specifically for this purpose, as detailed by Haggart et al. [15]. Our preprocessing pipeline began with the application of an exponential apodization function, which emulated a line-broadening of 0.5 Hz, followed by Fourier transformation. Subsequently, all spectra underwent reference alignment with respect to the glucose anomeric proton doublet (at ~ 5.23 ppm), automatic phasing, and baseline correction. After the initial alignment, we excluded both the water peak (4.4 ppm to 4.9 ppm) and a specified region associated with an added standard (− 0.04 to 0.04 ppm) to enhance data fidelity. The resulting data set comprised 28,119 intensities, which will be referred to as metabolomic features in the remainder.

For optimal normalization, these data were subjected to the probabilistic quotient method [16], which removed effects due to differences in total concentration between samples. Subsequently, final spectral alignment was performed utilizing the icoshift algorithm [17, 18]. This rigorous preprocessing regimen ensured the integrity and robustness of our dataset for subsequent analyses.

Among the 8814 individuals, 618 were excluded due to missing ADG phenotypes or genomic data. An additional 22 individuals were identified as outliers based on the metabolomic features and excluded following these steps: (1) principal component analysis (PCA) was performed on the metabolomic data of all individuals, (2) scores for the first and second principal components were obtained to determine each individual’s position, (3) the individual closest to the center of the distribution was designated as the reference, (4) pairwise distances between each individual and the reference were calculated, and (5) individuals with pairwise distances exceeding six standard deviations from the mean were classified as outliers.

### Statistical models and methods

#### GBLUP

GBLUP for ADG were computed by fitting the following model:

1 $$\mathbf{y}=\mathbf{X}\mathbf{b}+{\mathbf{Z}}_{g}\mathbf{g}+{\mathbf{Z}}_{p}\mathbf{p}+{\mathbf{Z}}_{l}\mathbf{l}+\mathbf{e},$$

where $\mathbf{y}$ is a vector of phenotypes for ADG, and $\mathbf{b}$ denotes a vector of fixed effects, including herd-section-year-month, birth parity, and regressions on initial weight and litter size at birth, $\mathbf{g}$ is a vector of additive genetic effects explained by SNPs, $\mathbf{p}$ is a vector of pen effects, $\mathbf{l}$ is a vector of litter effects, and $\mathbf{e}$ is a vector of residual effects, capturing variation that remain unexplained by the other model effects. Matrices $\mathbf{X}$, ${\mathbf{Z}}_{g}$, ${\mathbf{Z}}_{p}$, and ${{\varvec{Z}}}_{l}$ are corresponding incidence matrices for $\mathbf{b}$, $\mathbf{g}$, $\mathbf{p}$, and $\mathbf{l}$, respectively. Vectors $\mathbf{g}$, $\mathbf{p}$, $\mathbf{l},$ and $\mathbf{e}$ consist of random effects with $\mathbf{g}\sim N\left(0,\mathbf{G}{\sigma }_{g}^{2}\right)$, $\mathbf{p}\sim N(0,\mathbf{I}{\sigma }_{p}^{2})$, $\mathbf{l}\sim N\left(0,\mathbf{I}{\sigma }_{l}^{2}\right)$, $\mathbf{e}\sim N(0,\mathbf{I}{\sigma }_{e}^{2})$, and were assumed to be independent of each other. Matrix $\mathbf{G}$ is an additive genomic relationship matrix, computed using the VanRaden method 1 [10], $\mathbf{I}$ is an identity matrix, and ${\sigma }_{g}^{2}$, ${\sigma }_{p}^{2}$, ${\sigma }_{l}^{2}$, ${\sigma }_{e}^{2}$ are the variances of genetic, pen, litter and residual effects, respectively.

#### MGBLUP

The MGBLUP model used here aligns with Christensen et al. [7], where it was shown that EBVs in the metabolomic-genomic model can be obtained by solving two linear mixed model equation systems successively. In our context this implies that EBVs in the metabolomic-genomic model was obtained in two steps by successively applying MGBLUP_1_ and MGBLUP_2_, as described in the following.

The first step is:

2 $$\mathbf{y}=\mathbf{X}{\mathbf{b}}_{1}+{\mathbf{Z}}_{m}\mathbf{m}+{\mathbf{Z}}_{g}{\mathbf{g}}_{1}+{\mathbf{Z}}_{p}{\mathbf{p}}_{1}+{\mathbf{Z}}_{l}{\mathbf{l}}_{1}+{\mathbf{e}}_{1},$$

where$\mathbf{y}$,$\mathbf{X}$,${\mathbf{b}}_{1}$,${\mathbf{Z}}_{g}$,${\mathbf{g}}_{1}$,${\mathbf{Z}}_{p}$,${\mathbf{p}}_{1}$,${\mathbf{Z}}_{l}$,${\mathbf{l}}_{1}$, ${\mathbf{e}}_{1}$ are defined as for the GBLUP model, with subscript 1 referencing model MGBLUP_1_, and $\mathbf{m}$ is the vector of metabolomic effects on phenotype,$\mathbf{m}\sim N\left(0,\mathbf{Q}{\sigma }_{m}^{2}\right)$, with $\mathbf{Q}$ being the metabolomic similarity matrix defined as described in Guo et al. [11]. Let matrix $\mathbf{M}$ be a *p* × *q* matrix of NMR intensities with *p* = 8174 (equal to the number of samples) and *q* = 28,119 (equal to the number of metabolomic features), where columns of $\mathbf{M}$ were scaled to unit variance [19], then $\mathbf{Q}=\frac{\mathbf{M}{\mathbf{M}}^{\mathbf{^{\prime}}}}{q}$[18]. The distributions of random effects followed Christensen et al. [7]:${\mathbf{g}}_{1}\sim N\left(0,\mathbf{G}{\sigma }_{{g}_{1}}^{2}\right)$,${\mathbf{p}}_{1}\sim N\left(0,\mathbf{I}{\sigma }_{{p}_{1}}^{2}\right)$,${\mathbf{l}}_{1}\sim N\left(0,\mathbf{I}{\sigma }_{{l}_{1}}^{2}\right)$,${\mathbf{e}}_{1}\sim N\left(0,\mathbf{I}{\sigma }_{{e}_{1}}^{2}\right)$, with the different random effects assumed independent of each other.

The second step is:

3 $$\widehat{\mathbf{m}}=\mathbf{X}{\mathbf{b}}_{2}+{\mathbf{Z}}_{g}{\mathbf{g}}_{2}+{\mathbf{Z}}_{p}{\mathbf{p}}_{2}+{\mathbf{Z}}_{l}{\mathbf{l}}_{2}+{\mathbf{e}}_{2},$$

where $\widehat{\mathbf{m}}$ is the vector of predicted metabolomics effects from MGBLUP_1_ and other vectors and matrices are defined similar to those in MGBLUP_1_. The distributions of random effects were assumed ${\mathbf{g}}_{1}\sim N\left(0,\mathbf{G}{\sigma }_{{g}_{1}}^{2}\right)$, ${\mathbf{p}}_{1}\sim N\left(0,\mathbf{I}{\sigma }_{{p}_{1}}^{2}\right)$, ${\mathbf{l}}_{1}\sim N\left(0,\mathbf{I}{\sigma }_{{l}_{1}}^{2}\right)$, ${\mathbf{e}}_{1}\sim N\left(0,\mathbf{I}{\sigma }_{{e}_{1}}^{2}\right)$, ${\mathbf{g}}_{2}\sim N\left(0,\mathbf{G}{\sigma }_{{g}_{2}}^{2}\right)$, ${\mathbf{p}}_{2}\sim N\left(0,\mathbf{I}{\sigma }_{{p}_{2}}^{2}\right)$, ${\mathbf{l}}_{2}\sim N\left(0,\mathbf{I}{\sigma }_{{l}_{2}}^{2}\right)$, ${\mathbf{e}}_{2}\sim N\left(0,\mathbf{I}{\sigma }_{{e}_{2}}^{2}\right)$, with the different random effects assumed independent of each other, according to Christensen et al. [7], and based on the assumption of metabolomic features having the same variances.

For MGBLUP, the EBVs ($\widehat{\mathbf{a}}$) were calculated as the sum of EBVs from MGBLUP_1_ (${\widehat{\mathbf{g}}}_{1}$) and MGBLUP_2_ (${\widehat{\mathbf{g}}}_{2}$), following the derivation in Christensen et al. [7], i.e. $\widehat{\mathbf{a}}={\widehat{\mathbf{g}}}_{1}+{\widehat{\mathbf{g}}}_{2}$.

### Estimation of variance components

The full data set was used to estimate variance components in GBLUP and MGBLUP by restricted maximum likelihood (REML), using the DMU software package [20]. The calculation of parameters is as follows [21].

For GBLUP, ${\sigma }_{P}^{2}=(\overline{{G }_{d}}- \overline{G }){\sigma }_{g}^{2}+{\sigma }_{p}^{2}+{\sigma }_{l}^{2}+{\sigma }_{e}^{2}$, such that the heritability based on GBLUP is ${h}_{GBLUP}^{2}=(\overline{{G }_{d}}- \overline{G }){\sigma }_{g}^{2}/{\sigma }_{P}^{2}$, where $\overline{{G }_{d}}$ is the average of diagonal elements in the $\mathbf{G}$ matrix and $\overline{G }$ is the average of all elements of the $\mathbf{G}$ matrix, which is equal to 0.

For MGBLUP, the calculation is ${\sigma }_{{P}_{1}}^{2}=(\overline{{Q }_{d}}- \overline{Q }){\sigma }_{m}^{2}+(\overline{{G }_{d}}- \overline{G }){\sigma }_{{g}_{1}}^{2}+{\sigma }_{{p}_{1}}^{2}+{\sigma }_{{l}_{1}}^{2}+{\sigma }_{{e}_{1}}^{2}$, where $\overline{{Q }_{d}}$ is the average diagonal of the $\mathbf{Q}$ matrix, and $\overline{Q }$ is the average of the $\mathbf{Q}$ matrix, which is equal to 0. Here, the direct heritability is ${h}_{d}^{2}=(\overline{{G }_{d}}- \overline{G }){\sigma }_{{g}_{1}}^{2}/{\sigma }_{{P}_{1}}^{2}$, and the metabolomic variance ratio is ${c}_{m}^{2}=(\overline{{Q }_{d}}- \overline{Q }){\sigma }_{m}^{2}/{\sigma }_{{P}_{1}}^{2}$. Furthermore, according to Christensen et al. [7], heritability of the metabolomic intensities can be obtained from MGBLUP_2_, i.e. here ${h}_{m}^{2}=(\overline{{G }_{d}}- \overline{G }){\sigma }_{{g}_{2}}^{2}/[(\overline{{G }_{d}}-\overline{G }){\sigma }_{{g}_{2}}^{2}+{\sigma }_{{p}_{2}}^{2}+{\sigma }_{{l}_{2}}^{2}+{\sigma }_{{e}_{2}}^{2}]$ and, therefore, according to Christensen et al. [7], the total heritability based on the MGBLUP model is: ${h}_{MGBLUP}^{2}={c}_{m}^{2}{h}_{m}^{2}+{h}_{d}^{2}$.

### Accuracies of estimated breeding values

Prediction accuracies were estimated for two validation schemes. For each scheme, a validation population was defined and accuracies of EBVs of animals in the validation population were calculated when different information sources were available for the animals: (i) genotypes only, (ii) genotypes and metabolomic data, (iii) genotypes and phenotypic data, and (iv) genotypes, metabolomic data, and phenotypic data. The accuracies were estimated in three steps. First, we estimated predictive abilities of EBVs based on the partial dataset with genotypes on the validation population. Second, we estimated ratios of population accuracies based on partial (p) and whole (w) datasets using the LR method [12]. Third, we computed the actual population accuracies in the validation population by combining estimates predictive abilities with estimates of ratios of accuracies. These steps will be described in detail in the following, after a description of the two validation schemes.

#### Validation schemes

**Fig. 1:**
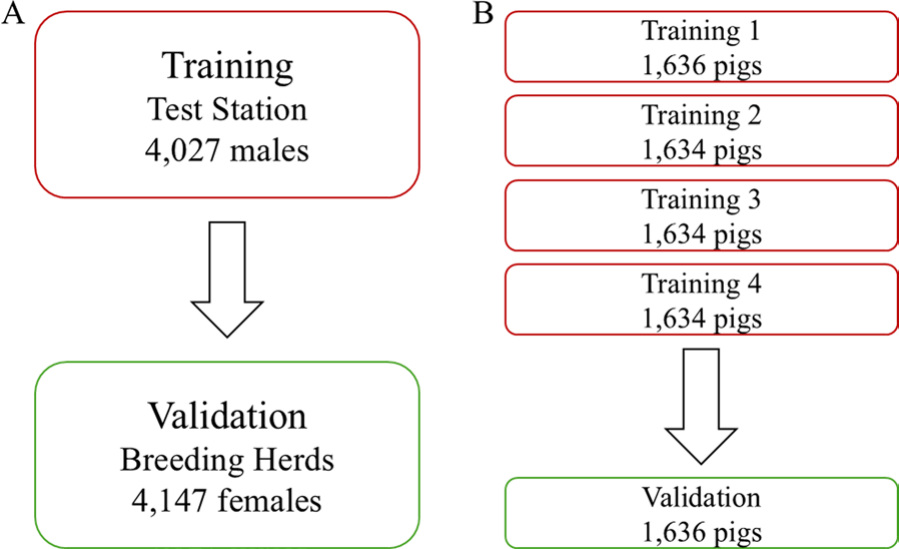
The two validation schemes. **a** Test station to breeding herds validation; **b** Five-fold cross-validation

The two validation schemes are shown in Fig. 1 and consisted of test station to breeding herds validation (Fig. 1a) and fivefold cross-validation (Fig. 1b). For the test station to breeding herds validation (TB), we partitioned the population into a training population consisting of male individuals from the test station and a validation population consisting of female individuals from breeding herds. This scheme enabled us to investigate the accuracy of EBV for individuals from breeding herds based on phenotypic data from individuals in the test station. The purpose of doing this was to mimic the situation when only phenotypic data from the test station is available, e.g. for feed efficiency, which is recorded at a test station because it is difficult/expensive to record. For fivefold cross-validation (5F), we randomly divided the entire population into five sub-populations of equal size. Each round of cross-validation involved designating four of the five sub-populations as the training population, with the fifth group serving as the validation population. This process was repeated for each sub-population, allowing us to assess the accuracy of the EBV of one sub-population based on phenotypic data from the remaining four sub-populations.

#### Predictive abilities

For both the TB and 5F validation schemes, the phenotypes, genotypes, and metabolomic features in the training population were included in the analysis and both phenotypes and metabolomic features were masked for the validation population. Breeding values of individuals in the validation population were predicted utilizing the data of the training population, alongside genotypes of individuals in the validation population.

To quantify the predictive ability of EBVs for individuals in the validation population, we computed the correlation between the vector of corrected phenotypes in the validation population and the vector of EBVs generated by GBLUP and MGBLUP. Corrected phenotypes are derived as the raw phenotypes minus the estimates of all fixed effects and covariates from a fixed effects model. Differences in predictive ability between GBLUP and MGBLUP were assessed using a Hotelling-Williams t-test [22, 23] at level 5%. For 5F, the final predictive ability score was computed as the average predictive ability across all five rounds.

#### Ratios of accuracies using the LR method

As described in previous studies [11, 12], the LR method provides estimators to assess EBVs based on partial (p) and whole (w) data sets. Here, for different scenarios of partial and whole data sets, as described below, we computed ratios of accuracies and dispersion biases based on vectors of EBVs for individuals in the validation population, ${\widehat{\mathbf{a}}}_{p}$ and ${\widehat{\mathbf{a}}}_{w}$ from partial and whole data sets, respectively.

The estimator of the ratio of accuracies is the correlation between ${\widehat{\mathbf{a}}}_{p}$ and ${\widehat{\mathbf{a}}}_{w}$, which is transformed into actual accuracy in the next section. The expected value of this estimator is the ratio of population accuracies from the partial and whole data sets, and this estimator therefore provides an assessment of the increase in population accuracy of EBVs when including the additional information in the whole dataset compared to the partial dataset, i.e. low correlation between ${\widehat{\mathbf{a}}}_{p}$ and ${\widehat{\mathbf{a}}}_{w}$ implies large increase in accuracy. The estimator of the dispersion is the slope of the regression of ${\widehat{\mathbf{a}}}_{w}$ on ${\widehat{\mathbf{a}}}_{p}$. The expectation of this estimator is 1, indicating no dispersion bias.

Partial and whole datasets were defined according to the data resources in the validation populations, where the scenario with more data resources is the whole data set and the scenario with less data resources is the partial data set. The scenarios were also named according to the data resources, i.e., GBLUPg is GBLUP with genomic data in the validation population, GBLUPgp is GBLUP with genomic and phenotypic data in the validation population, MGBLUPg is MGBLUP with genomic data in the validation population, MGBLUPgm is MGBLUP with both genomic and metabolomic data in the validation population, and MGBLUPgmp is MGBLUP with genomic, metabolomic and phenotypic data in the validation population.

#### Population accuracies of estimated breeding values

A measure of the population accuracy of EBVs for the validation data can be obtained by dividing the predictive ability by the square root of heritability. This accuracy is for the validation population in the situation where neither metabolomic and phenotypic data are available for these individuals, i.e. GBLUPg and MGBLUPg. Accuracy for GBLUPgp was obtained by dividing the accuracy of GBLUPg by the ratio of accuracies from GBLUPg and GBLUPgp,

i.e. ${\text{accuracy}}_{\text{GBLUPgp}} = \frac{{\text{accuracy}}_{\text{GBLUPg}}}{{\text{accuracy}}_{\text{GBLUPg}}/{\text{accuracy}}_{\text{GBLUPgp}}}$. Accuracies for MGBLUPgm and MGBLUPgmp were obtained similarly by dividing the accuracy of MGBLUPg by the ratios of accuracies from MGBLUPg and MGBLUPgm and from MGBLUPg and MGBLUPgmp, respectively.

The difference in accuracy between GBLUPg and MGBLUPg was assessed by testing whether the difference in predictive ability was statistically significant, as explained above. Differences in accuracies between other pairs of scenarios cannot be assessed by a statistical test due to the way the accuracies for GBLUPgp, MGBLUPgm and MGBLUPgmp were estimated.

## Results

In this study, we first estimated variance components and then computed EBVs using GBLUP and MGBLUP. For evaluation of these EBVs, we computed population accuracies based on two validation schemes and in scenarios with different data resources in the validation population.

### Estimates of variance components

**Fig. 2:**
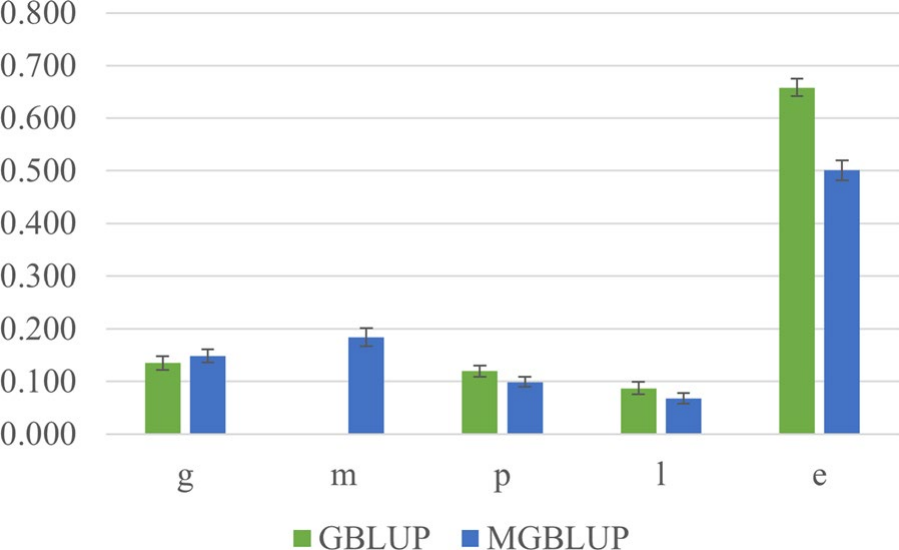
Ratio of estimates of variance components and their standard errors from different models. x-axis: variance components; y-axis: estimates of variances. g: ratio of variance of genomic effects; m: ratio of variance of metabolomic effects; l: ratio of variance of litter effects; p: ratio of variance of pen effects; e: ratio of variance of residual

Figure 2 shows the ratios of estimates of variance components to total phenotypic variances in the GBLUP and MGBLUP models (step 1). Additional file 1 Table S2 shows the summary statistics of the elements in matrices $\mathbf{G}$ and $\mathbf{Q}$ that were used to estimate the variance components, as explained in the methods section. The total phenotypic variances were 6507 g^2^ for GBLUP and 6338 g^2^ for step 1 of MGBLUP. The estimate of heritability was 0.14 for GBLUP and the estimates of direct heritability and proportion of variance explained by the metabolome were 0.15 and 0.18, respectively, for MGBLUP step 1. For MGBLUP step 2, the estimate of (common) heritability of metabolomics intensities was ${h}_{m}^{2}=0.14$ and, using the formula ${h}^{2}={c}_{m}^{2}{h}_{m}^{2}+{h}_{d}^{2}$, we obtain an estimate of heritability for MGBLUP equal to 0.17. Pen, litter and residual effects explained less variance when including metabolomic data into the model, which suggests that the metabolomic effects primarily capture some of the variance that was captured by these effects in the GBLUP model.

### Population accuracies of estimated breeding values

Estimates of population accuracies of EBVs in the validation population for GBLUP and MGBLUP are in Table 2; predictive abilities and method LR ratios of accuracies used to compute these accuracies are in Additional file 1 Tables S3 and S4, respectively. For the TB scheme, accuracies were 0.61 for MGBLUPg, 0.62 for MGBLUPgm, and 0.74 for MGBLUPgmp. Thus, the increase in accuracy when adding own metabolomic data was 6% of the increase when adding own phenotypes. For GBLUP, accuracies were 0.60 for GBLUPg and 0.72 for GBLUPgp, which are slightly lower than for, respectively, MGBLUPg and MGBLUPgmp. Accuracies for the 5F scheme were larger than for the TB scheme, at 0.87 for both MGBLUPg and MGBLUPgm, and 0.92 for MGBLUPgmp. Thus, the increase in accuracy when adding own metabolomic data was 10% of the increase when adding own phenotypes. For GBLUP, accuracies were 0.87 for GBLUPg and 0.91 for GBLUPgp. The difference between GBLUPg and MGBLUPg was statistically significant for TB but not for 5F. The estimators of the dispersion are in Additional file 1 Table S5, i.e. the slope of the regression of ${\widehat{\mathbf{a}}}_{w}$ on${\widehat{\mathbf{a}}}_{p}$, was close to 1 for the 5F scheme, indicating no dispersion bias, but equaled 0.82, 0.81, and 0.99 for the TB scheme for, respectively MGBLUPgmp on MGBLUPmg, MGBLUPgmp on MGBLUPg, and MGBLUPgm on MGBLUPg, indicating some dispersion bias for this scheme.

**Table 2:** Accuracy of estimated breeding values from different models

	GBLUPg	MGBLUPg	MGBLUPgm	GBLUPgp	MGBLUPgmp
TB	0.60	0.61	0.62	0.72	0.74
5F	0.87	0.87	0.87	0.91	0.92

TB: test station to breeding herd validation; 5F: fivefold cross-validation. GBLUP: genomic best linear unbiased prediction; MGBLUP: Metabolomic genomic best linear unbiased prediction with metabolomics similarity matrix. The lowercase letters, g, m and p refer to information included in the validation population, genomic (g), metabolomic (m), and phenotypic (p), respectively. For the TB scheme, the difference between GBLUPg and MGBLUPg is statistically significant

## Discussion

We hypothesized that for pigs without own phenotype, integrating own metabolomic data would yield an increase in accuracy of EBV of ADG, but results show that the increase in accuracy was only 6 and 10% of the increase in accuracy obtained when adding own phenotype for, respectively, the TB and 5F schemes.

Comparing parameter estimates, the estimate of heritability from GBLUP was 0.14, and for MGBLUP, the estimate of direct heritability was 0.15, that of the proportion of variance explained by metabolomics was 0.18, and that of the heritability of metabolomic features was 0.14, together resulting in a heritability estimate of 0.17. Altogether, this means that, although the metabolomic features are both heritable and capture some variation for the trait, most of the variation in the trait that is captured by the metabolomic features was environmental variation, and only a small proportion of the genetic variance was mediated by the metabolome.

Our estimate of heritability of 0.17 for ADG is similar to that from previous studies, e.g. estimates of 0.16 to 0.17 were found for Duroc pigs when applying animal models with or without spatial pen effects by Santiago et al. [24]. A slightly lower estimate of heritability of 0.13 was estimated using a model without spatial pen effects in Li et al. [25], but higher estimates were found by other studies, e.g. 0.32 by of Do et al. [26]. Therefore, the estimate of heritability in our study is within the range reported in the literature.

For the TB validation scheme, the accuracy of EBVs was larger for MGBLUP than for GBLUP, both when validation animals have own phenotype and when they do not. The difference in accuracy between GBLUPg and MGBLUPg was statistically significant (as mentioned in the methods section, a difference in accuracy between GBLUPgp and MGBLUPgmp cannot be assessed by a statistical significance test). For the 5F validation scheme, the accuracy for MGBLUPgmp was slightly larger than the accuracy for GBLUPgp, but the accuracies for MGBLUPg and GBLUPg were similar, and the latter difference was not statistically significant. As discussed above, in this study, the metabolomic features captured predominantly environmental variation in the trait. Therefore, the slightly larger accuracy for MGBLUP compared to GBLUP could be a consequence of a better modelling of the environmental variation by inclusion of metabolomic data. Investigating this more closely, we saw that the accuracies of the predicted direct breeding values, ${\widehat{\mathbf{g}}}_{1}$, from MGBLUP step 1 (results not shown), were the same as those for the EBVs, $\widehat{\mathbf{a}}$, suggesting that for this study, actually, we might ignore the genetic aspect of metabolomic data, and consider them to be environmental variables only.

For MGBLUP and both validation schemes, the accuracy when having own genomic and metabolomic data was almost identical to the accuracy when only having own genomic data, and smaller than the accuracy when having both own metabolomic data and own phenotype. These results are different from those of Guo et al. [11], where the accuracy increased substantially when incorporating own metabolomic data for all malting quality traits in barley, except for one, and was about 50% of the increase when including both own metabolomic data and own phenotype. However, in Guo et al.[11], the estimates of model parameters were also very different in nature from the current study, with the estimate of direct genetic variance in MGBLUP being substantially smaller than the estimate of genetic variance in GBLUP, and the proportion of estimated metabolomic variance in MGBLUP was very large, accounting for more than 50% of total phenotypic variance, together resulting in the estimate of the metabolome-mediated heritability being larger than that of the direct heritability. In the present study, the estimate of the metabolome-mediated heritability was small compared to the estimate of the direct heritability.

The increase in accuracy when incorporating own metabolomic data depends on the size of the metabolome-mediated heritability compared to the size of the direct heritability. The difference in traits and type of samples that were used to extract NMR profiles may also explain some of the differences in results between studies. While the NMR data from barley malt samples used in Guo et al. [11] reflect the metabolomic status at the time of sampling for malting quality traits, pig blood samples—collected at the end of the growth period—may not be representative of the full growth period. This is particularly relevant in the current study, where ADG was used as a model trait to evaluate the performance of MGBLUP. ADG reflects cumulative growth over time, whereas blood metabolomics provides only a snapshot of the physiological state at a specific time-point. Our results show that under this recording scheme, metabolomics data led to only a modest increase in EBV accuracy, possibly due to the mismatch between the time frame of the phenotype and the time of sample collection. Therefore, several biological and experimental factors likely contribute to the differences in prediction accuracies between our previous barley study and the current pig study.

In this regard, feed efficiency has the same characteristic as ADG and this suggests that we should also expect only a small increase in accuracy of EBVs for feed efficiency for these pigs. Increases in accuracy from MGBLUP may be different for other traits that are recorded in or over other time periods than ADG, such as meat quality traits or traits recorded on sows.

The model used in this study makes several assumptions that may influence results. The two-step method assumes equal variance components across metabolomic features, which simplifies computation but may obscure meaningful genetic variation. Variability in measurement precision, biological relevance, heritability, and environmental sensitivity among metabolomic features could result in diluted effects from influential features and amplified noise from less relevant ones. Exploring heritability estimates for individual metabolomic features could provide valuable insights, which could refine feature selection, enhance the accuracy of EBVs, and address limitations arising from the current model assumptions. We also assumed that the metabolomic effects are normally distributed and alternative models with mixture distributions may be worthwhile to explore in future work [27], but there could be computational challenges due to the need to use a Bayesian framework.

The above discussion highlights several potential avenues for future research, such as extending the model, investigating performance with blood sampling at multiple time points, metabolomics on samples of other tissues. Investigation of other traits is also needed.

## Conclusions

We investigated the inclusion of metabolomic data from blood samples taken at the end of test into genomic prediction models for pigs, using ADG as a model trait under a proper recording scheme. Results showed small increases in population accuracies of EBVs. We expect a similar performance for feed efficiency, since this trait is recorded over the same time period as ADG. Further work will be needed to investigate alternative time points for blood sampling, metabolomics on samples of other tissues, and other traits.

## Supplementary Information

Additional file 1.

## Data Availability

Data supporting this study are not publicly available due to data protection policy by private company. Please contact corresponding author for possibility.
