# Supplementary material for: Metabolomic-genomic prediction realizes small increases in accuracy of estimated breeding values for daily gain in pigs
Source: Genet Sel Evol. 2025 May 21;57:24. doi: 10.1186/s12711-025-00972-4 (PMC12096740; doi:10.1186/s12711-025-00972-4)
Supplement: Supplementary file 1 — Additional file 1. [file 12711_2025_972_MOESM1_ESM.docx]

**Metabolomic-genomic prediction realizes small increases in accuracy of estimated breeding values for daily gain in pigs**

Xiangyu Guo^1*^, Pernille Sarup^3^, Anders Bay Nord^4^, Mark Henryon^1^, Tage Ostersen^1^, Ole F. Christensen^1, 2^

^1^ Danish Pig Research Centre, Danish Agriculture & Food Council, 1609 Copenhagen V, Denmark

^2^ Center for Quantitative Genetics and Genomics, Aarhus University, 8000 Aarhus C, Denmark

^3^ Nordic Seed A/S, 8300 Odder, Denmark

^4^ Swedish NMR Centre, University of Gothenburg, Box 465, 40530 Göteborg, Sweden

^*^ Corresponding author:

Dr. Xiangyu Guo

[xguo@lf.dk](mailto:xguo@lf.dk)

E-mail addresses:

XG: xguo@lf.dk

PS: pesa@nordicseed.com

ABN: anders.bay.nord@nmr.gu.se

MH: mahe@lf.dk

TO: tao@lf.dk

OFC: olch@lf.dk

**Tables**

**Table S1.** Descriptive statistics for average daily gain.

| Trait^1^ | N-obs | Unit | Average | Variance | S.D. | Min | Max | PCV^2^ |
| --- | --- | --- | --- | --- | --- | --- | --- | --- |
| ADG | 8174 | g | 1254.66 | 11303.13 | 106.32 | 826.50 | 1564.60 | 8.47% |
| ADGm | 4027 | g | 1284.14 | 6886.01 | 82.98 | 1011.00 | 1524.00 | 6.46% |
| ADGf | 4147 | g | 1226.04 | 13932.05 | 118.03 | 826.50 | 1564.60 | 9.63% |
| ADGm20 | 1237 | g | 1290.37 | 6083.29 | 78.00 | 1055.00 | 1524.00 | 6.04% |
| ADGm21 | 2585 | g | 1280.78 | 7253.76 | 85.17 | 1011.00 | 1523.00 | 6.65% |
| ADGm22 | 205 | g | 1288.86 | 6758.71 | 82.21 | 1082.00 | 1509.00 | 6.38% |
| ADGf21 | 3850 | g | 1225.58 | 14204.36 | 119.18 | 826.50 | 1564.60 | 9.72% |
| ADGf22 | 297 | g | 1232.00 | 10399.82 | 101.98 | 962.40 | 1497.90 | 8.28% |

^1^ Trait: ADG = average daily gain.

^2^ PCV: coefficient of phenotypic variance

**Table S2.** The summary statistics of diagonal, off-diagonal values and all values in each matrix.

|  | Diagonal | | | | Off-Diagonal | | | | All | | | |
| --- | --- | --- | --- | --- | --- | --- | --- | --- | --- | --- | --- | --- |
| Matrix | Avg | SD | Min | Max | Avg | SD | Min | Max | Avg | SD | Min | Max |
| G | 1.005 | 0.060 | 0.818 | 1.261 | 0.000 | 0.060 | -0.238 | 0.753 | 0.000 | 0.061 | -0.238 | 1.261 |
| Q | 1.000 | 2.248 | 0.392 | 70.136 | 0.000 | 0.106 | -2.032 | 12.165 | 0.000 | 0.109 | -2.032 | 70.136 |

G: genomic relationship matrix; Q: metabolomic similarity matrix

**Table S3.** Predictive ability and accuracy of predicted breeding values.

|  | Predictive ability | | Accuracy | |
| --- | --- | --- | --- | --- |
| Model | **TB** | **5F** | **TB** | **5F** |
| GBLUPg | 0.213 | 0.306 | 0.605 | 0.867 |
| MGBLUPg | 0.217 | 0.306 | 0.615 | 0.867 |

GBLUP = genomic best linear unbiased prediction, MGBLUP = metabolomic-genomic best linear unbiased prediction; GBLUPg is GBLUP incorporating genotypes on individuals in validation population, MGBLUPg is MGBLUP incorporating genotypes on individuals in validation population.

TB: test station to breeding herd validation; 5F: 5-fold cross-validation.

**Table S4.** Ratios of population predictive ability of predicted breeding values using MGBLUP.

| Scheme | MGBLUPgmp/MGBLUPmg | MGBLUPgmp/MGBLUPg | MGBLUPgm/MGBLUPg | GBLUPgp/GBLUPg |
| --- | --- | --- | --- | --- |
| TB | 0.721 | 0.741 | 0.622 | 0.735 |
| 5F | 0.911 | 0.917 | 0.872 | 0.911 |

MGBLUP = metabolomic-genomic best linear unbiased prediction, GBLUP = genomic best linear unbiased prediction. gmp/gm is the ratio population accuracies on individuals in VP from MGBLUPgmp and MGBLUPgm, gmp/g is the ratio population accuracies on individuals in VP from MGBLUPgmp and MGBLUPg, gm/g is the ratio population accuracies on individuals in VP from MGBLUPgm and MGBLUPg, gp/g is the ratio population accuracies on individuals in VP from GBLUPgp and GBLUPg, where MGBLUPgmp is MGBLUP incorporating phenotypes, metabolomics and genotypes on individuals in VP, MGBLUPgm is MGBLUP incorporating metabolomics and genotypes on individuals in VP, and MGBLUPg is MGBLUP incorporating genotypes on individuals in VP, GBLUPgp is GBLUP incorporating phenotypes and genotypes on individuals in VP, and GBLUPg is GBLUP incorporating genotypes on individuals in VP.

TB: test station to breeding herd validation; 5F: 5-fold cross-validation.

**Table S5.** Dispersion bias from method LR.

| Scheme | MGBLUPgmp/MGBLUPmg | MGBLUPgmp/MGBLUPg | MGBLUPgm/MGBLUPg | GBLUPgp/GBLUPg |
| --- | --- | --- | --- | --- |
| TB | 0.822 | 0.812 | 0.988 | 0.834 |
| 5F | 1.008 | 0.997 | 0.990 | 1.010 |

MGBLUP = metabolomic-genomic best linear unbiased prediction, GBLUP = genomic best linear unbiased prediction. gmp/gm is the ratio population accuracies on individuals in VP from MGBLUPgmp and MGBLUPgm, gmp/g is the ratio population accuracies on individuals in VP from MGBLUPgmp and MGBLUPg, gm/g is the ratio population accuracies on individuals in VP from MGBLUPgm and MGBLUPg, gp/g is the ratio population accuracies on individuals in VP from GBLUPgp and GBLUPg, where MGBLUPgmp is MGBLUP incorporating phenotypes, metabolomics and genotypes on individuals in VP, MGBLUPgm is MGBLUP incorporating metabolomics and genotypes on individuals in VP, and MGBLUPg is MGBLUP incorporating genotypes on individuals in VP, GBLUPgp is GBLUP incorporating phenotypes and genotypes on individuals in VP, and GBLUPg is GBLUP incorporating genotypes on individuals in VP.TB: test station to breeding herd validation; 5F: 5-fold cross-validation.
